# Cost-Effective, Ester-Based Molecular Doping in Silicon

**DOI:** 10.3390/ijms26031024

**Published:** 2025-01-25

**Authors:** Anup Shrivastava, Jost Adam, Rosaria A. Puglisi

**Affiliations:** 1 Computational Materials and Photonics (CMP), Department of Electrical Engineering and Computer Science FB 16, University of Kassel, Wilhelmshöher Allee 71, 34121 Kassel, Germany; jost.adam@uni-kassel.de; 2 Institute of Physics FB 10, University of Kassel, Heinrich-Plett-Straße 40, 34132 Kassel, Germany; 3 Center for Interdisciplinary Nanostructure Science and Technology, University of Kassel, Heinrich-Plett-Straße 40, 34132 Kassel, Germany; 4 Consiglio Nazionale delle Ricerche (CNR) Istituto per la Microelettronica e Microsistemi (IMM), VIII Strada 5 Zona Industriale, 95121 Catania, Italy

**Keywords:** silicon, molecular doping, solar cells

## Abstract

When fabricating Si-based devices, many process steps require the use of expensive, high-power consumption, environmentally unfriendly, operator-unsafe machines, and processes. Among the many involved process steps, the ones needed to fabricate the metallurgical junction make use of conventional doping methods, which do not always represent optimal solutions. The high costs of the processing equipment and the use of hazardous materials, not to count the structural damage produced, intrinsically limit future developments towards nm-scaled and low cost approaches. Recently a chemistry-based method has been proposed to form the junction on Si, the so-called molecular doping. In this approach, the samples to be doped are subjected to a silylation process, during which a layer of dopant-containing molecules is deposited in a liquid bath kept at boiling temperature. After the coating, the samples are annealed to decompose the molecule and release the dopants inside the target. The peculiarity of using a liquid source allows for avoiding the structural damage. The entire doping procedure is simple and cost-effective, and it is based on the use of ester molecules, which are less harmful than the standard materials. In this work, we present experimental results on this chemistry-based technique, demonstrating its efficiency in creating the junction and demonstrate its feasibility in the fabrication of solar cells prototypes. Moreover, with respect to the literature, we show for the first time the effects of the protective layer presence over the dopant source molecules in the final solar cells electrical properties. As a proof of concept, we have numerically investigated the Si-based solar cell using the SCPAS-1D simulator. The finding claims that, the proposed samples have a good match in terms of the performance of the devices compared to the conventional Si-solar cells. Henceforth, the proposed work can provide a guideline to achieve less expensive, more environmentally friendly techniques for molecular doping in Si without affecting its performance in the metallurgical junction.

## 1. Introduction

Transitioning to sustainable semiconductor production is not anymore an option. Among the various semiconductor materials, Si has taken a leading role in electronic and photovoltaic applications due to its abundance, stability, and nontoxicity. Projections indicate that this dominance will continue in the coming years. However, industrial and societal demands for reduced material consumption and environment-friendly processes are driving the scientific and technological community to explore innovative solutions within the intriguing realm of nanostructures and ultra-thin films. Beyond miniaturization, the roadmap emphasizes low-cost and sustainable production methods. These requirements have posed constraints for the doping process, consisting in the introduction of the dopant impurities into the intrinsic semiconductor to modulate its electrical properties. Conventional doping methods, developed in the 1960‚ such as gas-phase processes or ion implantation‚ present cost and safety issues. Additionally, these methods need multiple and long processes to achieve abrupt doping profiles, conformal doping when the technology requires dopant atoms to follow three-dimensional nanostructured surfaces, channeling, charging effects, stochastic impingement of source atoms on the silicon surface, and crystal damage, essential when dealing with ultra-thin films. In the past, other approaches based on plasma physics, have also been proposed such as the plasma immersion ion implantation. This exhibited ultra-shallow diffusion depths, but it requires a deep understanding of the atomic processes taking place at the surface and considerable investments in terms of equipments [1]. The Molecular Doping (MD) has been proposed as an alternative, easy, and low-cost strategy to overcome the standard doping method drawbacks [2,3,4,5,6,7,8,9,10,11]. This method is based on the interaction between an organic molecule containing the dopant atom and the Si surface. The molecule is deposited through the immersion of Si in the solution containing the liquid precursors and forms a self-assembled layer on top of the target. The final surface density is correlated with the molecular steric footprint through a self-limiting process [2]. The sample decorated with the layer is then covered by silicon oxide for protection and is subjected to an annealing process that decomposes the molecule and diffuses the dopant atoms inside Si, with the kinetics dictated by the diffusion mechanisms. The protective layer, however, has been proven not be necessary, as the electrical performance of the material is still promising without it [12,13]. One of the main intrinsic advantages of MD is that it is based on a liquid source, which can then permeate the substrate of any structured surface, i.e. it provides conformality, and because of this advantage it could even be used in hollow nanostructured materials. The other positive aspect relies on the deterministic position of the source atoms, instead of stochastical as in the ion implantation or gas source based approach, because they are self-assembled on the Si surface in a packing configuration dependent on the steric properties of the chosen precursor molecule. The molecule assembling dynamics can also be controlled by changing the solution concentration, because it governs the molecules physisorption and chemisorption mechanisms [14]. Moreover, MD can be performed by using simple beakers and chemical reagents easy to be purchased and with costs of less than hundred dollars. Finally, the most important advantage is that the source dopant is deposited and released through damage-free processing, an essential requirement for its application to nanostructures and ultra-thin films. The method has been tested by using several precursors, annealing temperatures, or substrate types, demonstrating scalability, uniformity and reproducibility, and it has also been applied to FET-transistors and sensors [2,3,5,6,9,10,15,16,17]. In this paper, we present results regarding the fabrication process of a metallurgical junction using MD and an example of its application in Si solar cells. We investigate several processing conditions, such as covering the molecular layer with a sacrificial oxide layer, or not, as well as exploring different materials for the back contact. Since most of the literature on the MD doping focuses on the <100> orientation, we chose to explore its effects on the Si <111> substrates. This orientation allows for the vertical growth of silicon nanostructures by using bottom-up methods [18]. Knowing the effects of MD doping on this crystallographic orientation is important to set the reference when the doping is applied on nanostructures. Moreover, Si <111> surfaces provide a well-defined model system to derive atomistic mechanisms [12]. We investigate the electrical properties of the solar cells under standard solar conditions, and in reverse polarization in order to understand the contribution of the parasitic resistances and to predict the potentiality of our proposed approach [19]. We finally performed numerical simulation of the solar cells, in order to explore, as a proof of concept, the potential limitations of the proposed approach compared with a standard silicon solar cell processing. To investigate the performance of the proposed solar cell, we performed a numerical simulation using SCAPS-1D.

## 2. Materials and Methods

### 2.1. Device Fabrication

We cut a p-type Si wafer with a <111> crystallographic orientation and 5–10 ohm/cm resistivity into 1 × 1 cm^2^ size pieces. Before the MD process, we cleaned the samples in acetone, alcohol, and water, and then etched them with a 16% HF solution for 60 s to remove the native silicon oxide. Immediately after etching, we immersed the samples in a solution of 20% (*v*/*v*) diethyl-l-propyl phosphonate and mesitylene at approximately 160 °C for 2.5 h. This step created a precursor layer over the Si surface. A group of samples were covered by a sacrificial layer made of 100 nm thick SiO_2_ spin on a glass (SOG) cap layer. To proceed with the cap layer deposition, we baked the samples at 80 °C for 60 s on a hot plate and then deposited the SOG oxide by spin coating at 3000 rpm for 20 s. A subsequent baking step at 250 °C for 60 s in air and a final annealing step at 425 °C for 1 h under 2 L/min flow of $N_{2}$ were performed to solidify the SOG layer. All of the samples, with and without the cap layer, were annealed in a furnace at 1050 °C for 500 s in a N_2_ flux (2 L/min) to decompose the precursor and diffuse and activate the dopant. We removed the parasitic junction on the back and then realized the back contact through two different routes: (i) 200 nm aluminum deposited by sputtering and heated in a furnace at 500 °C; and (ii) silver paste deposition. We then removed the native oxide layer on top of the first group, and the 100 nm thick sacrificial layer on top of the second group of samples. We then formed the top metallic contact by photolithography, which defined a finger bus geometry with eight stripes 200 μm large and a pitch of 800 μm, and performed the subsequent sputtering deposition of nickel and gold at a thickness of 160 nm. No passivation process was performed on the samples, neither anti-reflection film deposition. The carrier concentration was measured by spreading resistance profiling (SRP) on the control samples after the thermal annealing. We acquired the current–voltage (IV) characteristics in the dark and under controlled light conditions using a Keithley 237 source-measure unit. The light irradiation was pursued using a solar simulator based on a 150 W Xenon lamp, with a light spot of 3.5 cm and equipped with an ASTM filter that produces the AM1.5G solar spectrum. The collimated beam of the lamp was directed normally to the surface of the sample, fully covering the whole sample area in all of the cases. The light intensity measurement was performed using a calibrated Si photodiode.

### 2.2. Shockley Equation Fit

To determine the cells’ performance parameters, we fitted the experimental data to the well-known Shockley equation

(1) $$\begin{array}{c} I=I_{\mathrm{ph}}-I_{0}\left(\exp\left(\frac{q(V+R_{s}I)}{nk_{B}T}\right)-1\right)-\frac{V+R_{s}I}{R_{\mathrm{sh}}}, \end{array}$$

where $I_{\mathrm{ph}}$, $I_{0}$, $R_{s}$, $R_{\mathrm{sh}}$, *q*, *n*, $k_{B}$, and *T* are the photocurrent, saturation current of the diode, series resistance, shunt resistance, electron charge, ideality factor, Boltzmann constant, and temperature, respectively. The series resistance $R_{s}$ represents the total accumulated cell resistance, comprising the interfacial and active layer resistances, the electrode resistances, and contact and interconnect resistances [20]. The shunt resistance $R_{\mathrm{sh}}$ is due to leakage across the pn-junction around the edge of the cell, and the presence of crystal defects and/or impurities in the junction region [19,21]. Due to the implicit and non-linear nature of Equation (1), we employed non-linear least-squares fitting, based on the Levenberg–Marquardt algorithm [22,23,24], and implemented into the freely available Python library lmfit [25].

## 3. Results and Discussion

**Figure 1:**
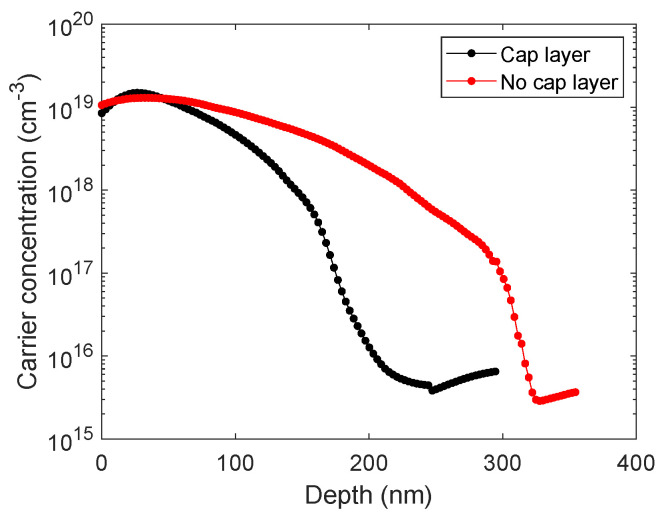
Electrically active carrier concentration as a function of the Si depth obtained by MD on planar wafers for the samples protected by the oxide cap layer (black symbols) and without protection layer (red).

After performing the MD process, we characterized the electrical properties of the doped planar control samples. The concentration profile of the electrically active carriers, shown in Figure 1, exhibited a peak of about $1.48\times 10^{19}$ cm^−3^ and $1.3\times 10^{19}$ cm^−3^, respectively for the case with the cap layer (black curve) and without (red), and a diffusion depth of 250 and 330 nm. The electrically active dopant atoms calculated from the experimental profiles were $1.18\times 10^{14}$/cm^2^ and $1.65\times 10^{14}$/cm^2^.

The higher doping efficiency found for the no-cap layer condition is in line with the literature [13]. This can be attributed to the fact that, during annealing, the molecules combine and diffuse preferably towards the SOG oxide side rather than towards the Si substrate. If we then take the no-cap case as a reference and compare it with the theoretical maximum dopant dose obtainable, we find that the DPP molecular footprint estimated through numerical modeling (using Avogadro molecular editor) is 0.49 nm^2^; the DPP surface density in the hypothesis of maximum molecular packing is then 2.04 × 10^14^ cm^−2^. The doping yield, taken as the ratio between the dose calculated from the SRP profile and the largest packable molecule density, is 80.8%. However the interactions with the Si substrate must also be taken into account. In Si <111>, the crystallographic orientation of the SRP planar samples presents a density of 7.83 × 10^14^ cm^−2^ of dangling bonds, which means that the maximum density that the molecule can form in the tri-dentate case is 1/3, i.e., 2.61 × 10^14^ cm^−2^ [11,26]. The doping yield, calculated as the ratio between the SRP dose and the maximum number of molecules bonded to the substrate, is 63%. This result could be ascribed to the interstitial carbon atoms, $C_{i}$, coming from the molecule, which can bond with substitutional phosphorus, $P_{s}$, forming the pair $C_{i}-P_{s}$. These pairs have been linked to multiple deep energy levels that contribute to the P electrical deactivation [27].

**Figure 2:**
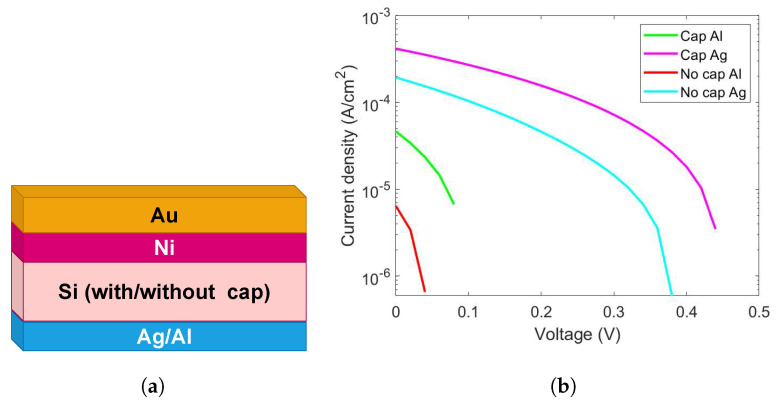
(**a**) Schematics for the simulated solar cell configuration. The front electrode is created using 160.00 nm thick Au and Ni layers. Two alternative types of back contacts (Al or Ag) have been used with distinct samples in both cases. (**b**) Electrical characterization at 1 Sun for the solar cells fabricated using MD doping, with the protective cap layer, for the case with the Al (green) and Ag (magenta) back contact, and without the cap, Al (red) and Ag (cyan).

Figure 2 reports the electrical characterization results, acquired at 1 Sun, obtained for the cells fabricated using MD doping, with and without the protective cap layer, with Al or Ag as back contacts.

The results show that the silver contact provides a better electrical performance in terms of I_sc_ and V_oc_, so the following discussion refers to cells fabricated using this type of metallic contact. Specifically, we wanted to investigate the electrical behavior of the devices under reverse polarization in order to evaluate the contribution of parasitic resistance and to understand the potentiality of our proposed technology.

**Figure 3:**
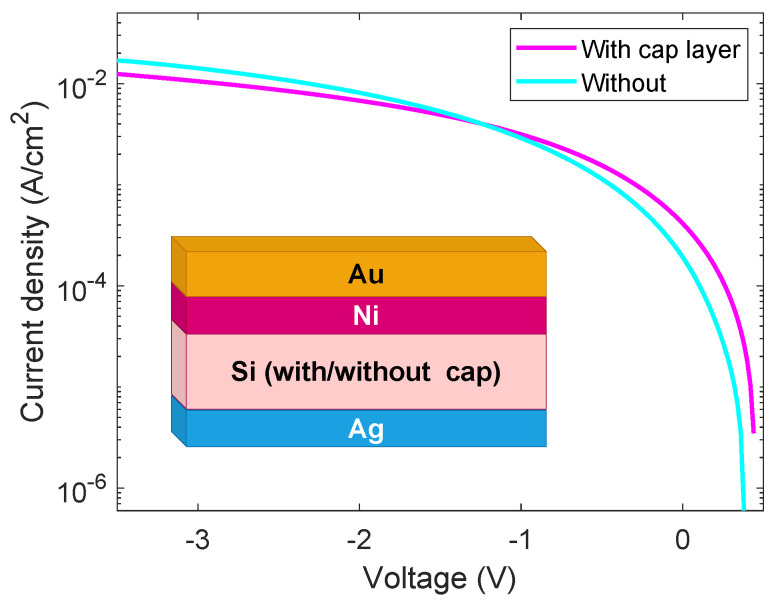
Comparison between the solar cells fabricated by MD doping, with (magenta) and without (cyan) the protective cap layer, and Ag back contact, characterized under reverse polarization.

Figure 3 shows the results of this investigation, performed under reverse polarization, on devices with Ag back contact and where the junction was fabricated with (magenta) and without (cyan) the protective cap layer. As it is clearly visible, the large parasitic resistances produce a low fill factor, equal to FF = 0.16% for the cap-layer cell, which is in turn affecting the $J_{SC}$, 0.37 × 10^−3^ A/cm^2^, and the whole efficiency of the prototype, equal to 0.03%. The low FF and efficiency values can be attributed to the shadowing and resistive effects of the opaque metallic contact, which are not optimized for this type of technology, and to the absence of passivation processes. These parasitic effects also impact I_sc_, which is related to the shunt and series resistances. It is important to estimate the effect of the parasitic resistances to understand the possible role of the MD doping.

### Fitting Results

**Figure 4:**
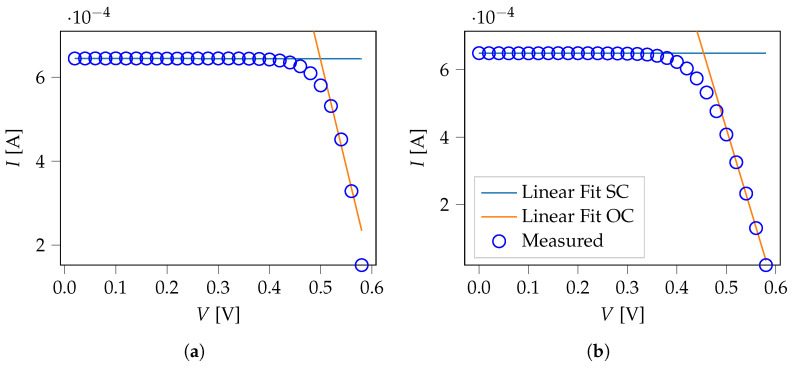
Experimental data and initial slope fits for (**a**) Cell 1 and (**b**) Cell 2.

We first performed a linear regression on the experimental *I*–*V* data to determine initial values for the $R_{\mathrm{sh}}$ and $R_{s}$ values. We determine our initial resistance values via $R_{\mathrm{sh}}=-1/\delta_{\mathrm{SC}}$ and $R_{s}=-1/\delta_{\mathrm{OC}}$, with the fitted slopes $\delta_{\mathrm{SC}}$ and $\delta_{\mathrm{OC}}$. Figure 4 demonstrates the experimental data alongside the slope fits.

With the once-determined initial values for $R_{\mathrm{sh}}$ and $R_{s}$, we fitted Equation (1) to the experimental data to gain the remaining intrinsic factors for the cells at hand (Table 1). We calculated the fit assuming a constant temperature of 25.00 °C, so the optimization parameters are $I_{\mathrm{ph}}$, $I_{0}$, $R_{s}$, $R_{\mathrm{sh}}$, and *n*.

## 4. Solar Cell Simulation (Proof-of-Principle)

As a proof of concept, we numerically simulated the simplest solar cell by using both fabrication procedures (with and without the sacrificial capping layers).

To investigate the performance of the proposed solar cell, we performed a numerical simulation using SCAPS-1D [28,29], which solves fundamental semiconductor equations such as drift-diffusion, Poisson’s equation, and continuity equations, as follows:

(2) $$\frac{\partial }{\partial x}\left(\varepsilon_{0}\varepsilon_{r}\frac{\partial \phi }{\partial x}\right)=-q\left(p-n+N_{D}^{+}-N_{A}^{-}+\frac{\rho_{def}}{q}\right)$$

(3) $$-\frac{\partial J_{n}}{\partial x}-U_{n}+G_{n}=\frac{\partial n}{\partial t}$$

(4) $$-\frac{\partial J_{p}}{\partial x}-U_{p}+G_{p}=\frac{\partial p}{\partial t}$$

where $J_{p}=-\frac{\mu_{p}p}{q}\frac{\partial E_{F_{p}}}{\partial x}$ and $J_{n}=-\frac{\mu_{n}n}{q}\frac{\partial E_{F_{n}}}{\partial x}$ are the current densities of holes and electrons, respectively. The symbols $\epsilon_{0}$, $\epsilon_{r}$, ψ, q, n, and p designate the absolute and relative permittivity, electrostatic potential, electronic charge, electron and hole concentration, respectively. The charge carrier mobilities, generation, and recombination rates for the electrons and holes are represented by $\mu_{n}$, $G_{n}$, $U_{n}$, $\mu_{p}$, $G_{p}$, $U_{p}$, respectively. $E_{Fn}$ and $E_{Fp}$ are used for the electrons and holes Fermi-level. All of these parameters are a function of the position coordinate ‘*x*’. During the numerical simulations, we assumed that the reflection from the top surface was zero, while the reflection from the back surface was 100%. This means that the entire incident photon had more energy than the bandgap of the materials and could contribute to the generation of the charge carriers.

The generated charge carrier mobility of the chosen material assisted in delivering it to the contacts. The schematic structure of the proposed configurations is the one reported in Figure 2a, where we numerically investigated four different structures corresponding to our experimental samples, with a bi-layer of Au and Ni as the top electrode. Under front contact, the Si bulk where the junction is created by MD is visible for the two explored cases—with or without a cap layer. Ag and Al are used as back contact as two different cases, as discussed in the Section 2.1. The performance of solar cells prominently depends on the layer structure, work functions of back and top electrodes, layer thickness, parasitic resistance (series and shunt), etc. We optimized our theoretical model based on the standard simulation parameters for conventional Si and the experimental results derived for the molecular-doped samples. The work functions for the front and back electrodes (Au, Al, and Ag) were taken from the literature [30]. The work functions for the metallic contacts were in the following ranges: Au 5.10 eV–5.47 eV, Al 4.06 eV–4.26 eV, and Ag 4.26 eV–4.71 eV. We simulated a thin layer of Ni (80.00 nm), under the Au (80.00 nm), to replicate the experimental conditions, as discussed in the synthesis section. The Ni is used to provide a proper adheshion to the Au electrode and make better contact. We did not observe any significant impact of this layer on the device performance.

**Figure 5:**
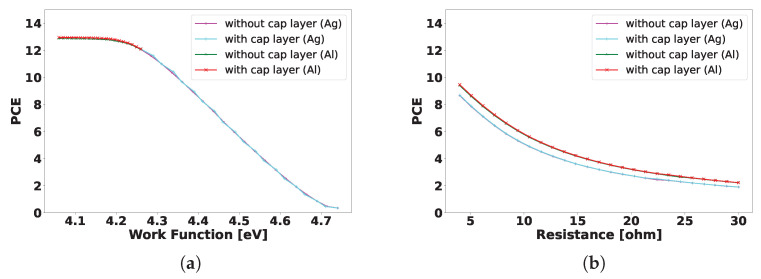
(**a**) PCE vs work function (**b**) PCE vs. series resistance, with and without capping layers, and using Ag or Al as back contact under the illumination of the AM1.5G solar spectrum.

Taking all of the other simulation parameters to be standard, we simulated the proposed structures under the illumination of the AM1.5 (1-Sun) solar spectrum. The key findings for all four sets of samples are summarized in Figure 5a,b.

Figure 5a demonstrates the variation of PCE versus the work function of the back electrode in the case of Si with and without the sacrificial cap layer.

The figure confirms the validity of our approach, as it can be observed that the sample behavior does not differ from the projected silicon standard case. A variation in PCE with parasitic resistance for all configurations is shown in Figure 5b. The results follow an expected trend as with lowering the series resistance, the power conversion efficiency can be increased. Also, the samples with Al as a back contact exhibited a slightly better performance in the case of the Ag back contact samples. It is important to mention that the simulations of the devices based on molecular doping follow the same behavior, as of a conventional Si cell. This can suggest that the proposed ester-based molecular doping technique will not affect the performance of a device, while providing a cost-effective, environmentally friendly and easy-to-integrate approach.

## 5. Conclusions

In this paper, we present results on the fabrication process of a metallurgical junction using MD and an give an example of its application to Si solar cells. We investigated several doping conditions, such as covering the molecular layer with a sacrificial oxide, or not, and explore different materials for the back contact. The results of the doping processes demonstrate the concentration profiles of the electrically active carriers with peaks as high as $1.48\times 10^{19}$ cm^−3^, with doses of 1.18–1.65 × 10^14^/cm^2^. The results are in line with the literature. We then integrated the MD doping process in silicon solar cell prototypes, with different back contacts—Ag or Al. We also investigated the electrical behavior of the devices under reverse polarization so as to evaluate the contribution of the parasitic resistance to understand the potentiality of the proposed technology. The low FF and efficiency values are attributed to the shadowing and resistive effects of the opaque metallic contact, which are not optimized for this type of technology, and to the absence of passivation processes. To investigate the performance of the proposed solar cell, as a proof of concept, we numerically simulated the solar cell using SCAPS-1D by taking both fabrication procedures into account (with and without the sacrificial capping layers). From the simulations, it is understood that the MD Si samples follow a similar behavior as in the standard silicon solar cells. This indicates that the proposed ester-based molecular doping technique did not affect the electrical performances/characteristics of the cells, while ensuring a cost-effective, environment-friendly, and easy to synthesize alternative to standard doping methods.

## Figures and Tables

**Table 1:** Intrinsic cell parameters determined by the non-linear least-squares fit concerning Equation (1), and the experimental data displayed in Figure S1a,c (Cell 1) and Figure S1b,d (Cell 2).

Cell	$I_{\mathrm{ph}}$ (A)	$I_{0}$ (μA)	$R_{s}$ (Ω)	$R_{\mathrm{sh}}$ (Ω)	*n*	$\chi^{2}$
1	6.45 × 10^−4^	6.84 × 10^−12^	32.57	6.04 × 10^12^	1.26	3.94 × 10^−12^
2	6.49 × 10^−4^	1.78 × 10^−11^	123.55	4.12 × 10^13^	1.30	7.15 × 10^−12^

## Data Availability

Data are contained within the article and Supplementary Materials.

## Supplementary Materials

The supporting information can be downloaded at: https://www.mdpi.com/article/10.3390/ijms26031024/s1.
