# Supplementary material for: Cost-Effective, Ester-Based Molecular Doping in Silicon
Source: Int J Mol Sci. 2025 Jan 25;26(3):1024. doi: 10.3390/ijms26031024 (PMC11818006; doi:10.3390/ijms26031024)
Supplement: Supplementary file 1 [file ijms-26-01024-s001.zip › SI.pdf]

International Journal of
Molecular Sciences

MDPI

Article

Cost-effective, ester-based molecular doping in silicon.

Anup Shrivastava [2], Jost Adam [2,3], and Rosaria A. Puglisi [1,*]

[1] Consiglio Nazionale delle Ricerche (CNR) Istituto per la Microelettronica e Microsistemi (IMM), VIII Strada 5 Zona Industriale, 95121 Catania, Italy;
[2] Computational Materials and Photonics (CMP), Department of Electrical Engineering and Computer Science FB 16 and Institute of Physics FB 10, University of Kassel, Wilhelmshöher Allee 71, 34121 Kassel, Germany;
[3] Center for Interdisciplinary Nanostructure Science and Technology, University of Kassel, Heinrich-Plett-Straße 40, 34132 Kassel, Germany

1. Supplementary Information

1.1. Non-linear fitting curves

Figure S1 shows the nonlinear fit results, alongside the point-wise fitting errors. Table 1 lists the resulting fitting parameters and the corresponding χ^2 accumulated error values, indication an excellent fit.

check for updates

Received:
Accepted:
Published:

Citation: Puglisi, R.A.; Adam, J. A chemistry-based technique to perform silicon doping. *Int. J. Mol. Sci.* **2021**, *1*, 0. https://doi.org/

Copyright: © 2020 by the authors. Licensee MDPI, Basel, Switzerland. This article is an open access article distributed under the terms and conditions of the Creative Commons Attribution (CC BY) license (https://creativecommons.org/licenses/by/4.0/).

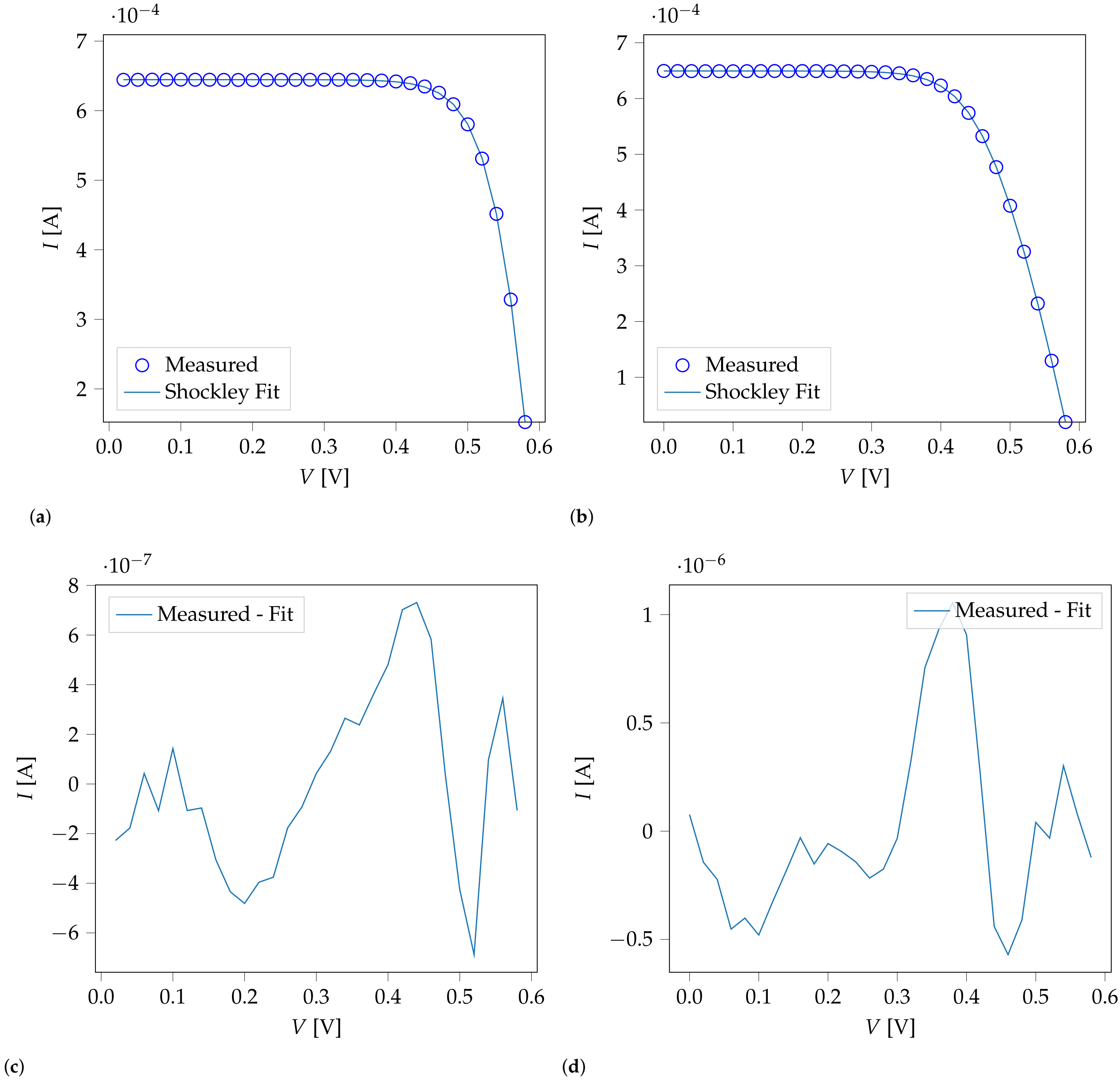

Figure S1. Non-linear least-squares fit and respective point-wise errors for Cell 1 ((a) and (c)), and Cell 2 ((b) and (d))
