# Supplementary figures and images for: Cost-Effective, Ester-Based Molecular Doping in Silicon
Source: Int J Mol Sci. 2025 Jan 25;26(3):1024. doi: 10.3390/ijms26031024 (PMC11818006; doi:10.3390/ijms26031024)

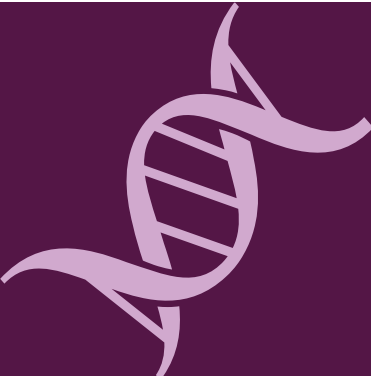

International Journal of  
*Molecular Sciences*

Supplement: Supplementary file 1 [file ijms-26-01024-s001.zip › Definitions/ijms-logo-eps-converted-to.pdf]

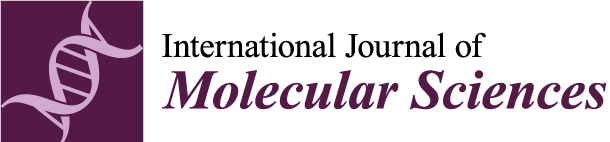

Supplement: Supplementary file 1 [file ijms-26-01024-s001.zip › Definitions/ijms-logo.png]

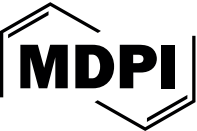

Supplement: Supplementary file 1 [file ijms-26-01024-s001.zip › Definitions/logo-mdpi-eps-converted-to.pdf]

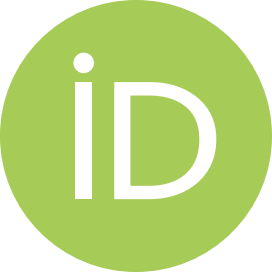

Supplement: Supplementary file 1 [file ijms-26-01024-s001.zip › Definitions/logo-orcid-eps-converted-to.pdf]

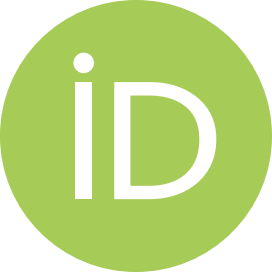

Supplement: Supplementary file 1 [file ijms-26-01024-s001.zip › Definitions/logo-orcid.pdf]

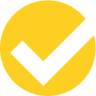

check for  
updates

Supplement: Supplementary file 1 [file ijms-26-01024-s001.zip › Definitions/logo-updates-eps-converted-to.pdf]

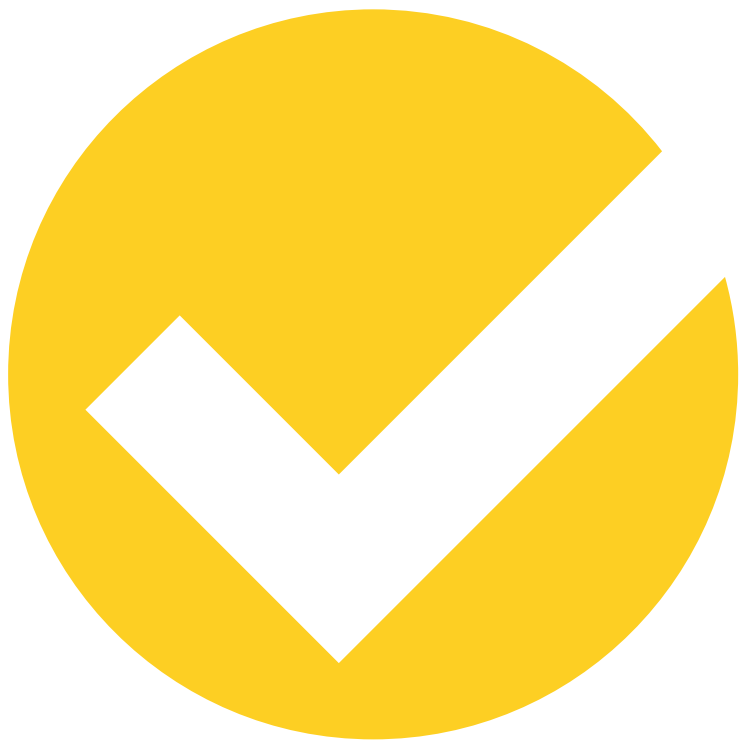

check for  
updates

Supplement: Supplementary file 1 [file ijms-26-01024-s001.zip › Definitions/logo-updates.pdf]
